# Treatment of arterial calcification in patients with chronic limb threatening ischemia with etidronate: protocol of an investigator-initiated multicenter, double blind, placebo-controlled, randomized clinical trial

**DOI:** 10.1186/s42155-022-00298-y

**Published:** 2022-06-06

**Authors:** R. Hoogervorst, H. van Overhagen, P. A. de Jong, W. Spiering, G. J. de Borst, H. T. C. Veger, A. T. A. Mairuhu, W. P. T. M. Mali

**Affiliations:** 1 Haga Hospital, HagaZiekenhuis, The Hague, The Netherlands; 2 UMC Utrecht, The Hague, The Netherlands

**Keywords:** Etidronate, CLTI, Arterial calcification

## Abstract

**Background:**

Pathologic studies have shown that in patients with critical limb threatening ischaemia (CLTI) medial arterial calcifications are frequently found and may be responsible for aggravating the disease. These extensive calcifitcations are found not only in arteries of the leg but also in the coronary arteries and the aorta. The progression of these calcifications is fast and they stiffen the vessel wall and may thus increase the cardiovascular risk.

Reduction of progression of calcification may not only reduce the burden of CLTI but may also reduce the high residual cardiovascular risk. Medial calcifications have been halted by etidronate in other trials. Its potential to reduce the burden from peripheral vascular disease in CLTI and residual cardiovascular risk remains to be established.

**Methods:**

This is an investigator-initiated multicenter, double blind, placebo-controlled, randomized trial comparing the effects of etidronate versus placebo in patients with CLTI.

Subjects will be randomized to either treatment with etidronate for 12 months (cyclical 20 mg/kg for 2 weeks on and 10 weeks off) orally or placebo for 12 months (in a similar routine).

The primary endpoint is the change in arterial calcification as quantified by CT-scan.

Secondary endpoints are the number of amputations above and below the ankle**,** mortality, number of vascular interventions and quality of life.

**Discussion:**

Up to now, the inert end stage of vascular disease in patients with CLTI, has been considered calcification of vessel walls. We believe there is reason to reverse causation and hypothesize that calcification causes vascular disease. This reversal can be proven in a clinical trial if halting the calcification process improves the outcome of the patient. Therefore we use etidronate, a bisphosphate that has proven to stop the calcification in several rare monogenetic calcifying diseases. We aim to perform this mechanistic proof-of-concept study hopefully leading to a clinical outcome study later on.

## Background

CLTI is the most severe type of peripheral vascular disease. Patients with CLTI have pain at rest and skin wounds in the leg(s). In addition, the cardiovascular mortality is high. A recent systematic review and meta-analysis on the outcome of conservative treatment of CLTI patients showed that within 1 year of diagnosis 18% of the patients died and 27% underwent amputation. The one year amputation free survival rate was 60% (Spreen et al. 2016; Van Reijen et al. 2021). In addition, the PADI-trial reported a 10-year survival of only ±20% (Konijn et al. 2020b).

With an estimated yearly incidence of 500–1000 new cases per million individuals in Western society, CLTI poses a considerable burden on patients’ health and resources (Teraa et al. 2016).

Several histopathologic studies of amputated legs of CLTI patients have shown that calcification of the media is present in up to 72% of patients and that the classical atherosclerotic disease is less pronounced (O’Neill et al. 2015; Soor et al. 2008) In a group of 54 CLTI patients, we reported that virtually all had severely calcified peripheral vessels, coronary arteries and a severely calcified aorta. The median coronary artery calcium score was 1485 and in 19 of 45 (35%) patients, it was above 2000. The thoracic and the abdominal aorta where completely annular calcified in 37%, 31% of cases respectively.

Although treatment of CLTI has improved, there still is a high cardiovascular mortality. Current treatment strategies target on luminal thrombosis and cholesterol-driven atherosclerosis, a process mainly located in the intima. However, a process which co-occurs with atherosclerosis, arterial calcification, may independently lead to vascular disease. Arterial calcifications, especially when located in the media, are a cause of vascular stiffening and an independent cause of vascular disease (Konijn et al. 2021; Konijn et al. 2020a; Lanzer et al. 2014; Teraa et al. 2016) A meta-analysis showed that calcification in any vascular bed is associated with a three to fourfold increased risk for cardiovascular events and mortality (Rennenberg et al. 2009).

We postulate that medial arterial calcification can aggravate CLTI and at the same time can be present in other vascular territories causing acute coronary syndrome or stroke. Halting the progression of these calcifications may improve the outcome of the patients.

The strongest inhibitor of vascular calcification in the body is inorganic pyrophosphate (PPi) (Evrard et al. 2015; Fleisch et al. 1965). Etidronate, the oldest bisphosphate is a stable PPi analogue, which has been used for the treatment of osteoporosis. It stops the vascular calcification in several rare monogenetic calcifying diseases and was effective in several RCT’s (Bartstra et al. 2020; Kawahara et al. 2013; Kranenburg et al. 2018; Oliveira and Oliveira 2016) A meta-analysis showed that the risk of cardiovascular mortality in patients treated with bisphosphonates decreased with 19% (RR 0.81; 95% CI 0.64–1.02) and that the risk of all-cause mortality decreased with 10% (RR 0.90; 95% CI 0.84–0.98) (Kranenburg et al. 2016).

Although there is evidence that etidronate is effective in improving proxy endpoints, both mechanistic studies and studies with clinically relevant endpoints are lacking in CLTI.

## Methods/design

This is an investigator-initiated double blind, placebo-controlled, randomized clinical trial comparing the effects of etidronate (*n* = 40) versus placebo (*n* = 40), taking a 25% dropout in to account, in patients with CLTI.

Our objective is to quantify the effects of etidronate on CT measured arterial calcification in patients with CLTI.

### Study population

Seventy-six patients with CLTI, stable for 2 weeks, will be recruited. After the screening for eligibility and explanation of the potential benefits and risks, the participants will sign the informed consent.

### Inclusion criteria

- ≥55 years old and stable CLTI for at least two weeks
- CLTI is defined as Fontaine stage 3 (rest pain and pain at night) or 4 (tissue loss)

### Exclusion criteria

- eGFR < 30 ml/min/1.73 m2
- Osteomalacy
- abnormality of the esophagus interfering with the passage of the drug
- Use of bisphosphonates during the last 5 years

### Intervention and comparator

Subjects will be randomized to either treatment with etidronate orally for 12 months (cyclical 20 mg/kg for 2 weeks on and 10 weeks off) or placebo for 12 months (in a similar cyclical routine).

### Outcome parameters

*Primary outcome*: changes in arterial calcification in a total vascular score as quantified by CT-scan at baseline,6 months and at 1 year. Calcification will be quantified in the intracranial and extracranial carotid arteries, coronary arteries, thoracic and abdominal aorta and iliac, femoral and crural arteries. CT calcium volume will be quantified using dedicated applying a threshold of 130 Hounsfield Units for calcium.

*Secondary outcomes***:** numbers of amputations above and below the ankle, mortality, number of vascular interventions, quality of life as measured with the short form 36 (SF-36) health survey, serum calcium and phosphate and inorganic pyrophosphate.

### Sample size calculation and data analysis

We used data from the observational cohort study DIACART with type 2 diabetes patients with peripheral vascular disease, at least a history of cardiac disease, aged > 55 (Bourron et al. 2020). This cohort resembles our CLTI patient group as shown in the pooled data from our JUVENTAS and PADI studies in witch there were 281 CLTI patients included of whom 49.1% had diabetes type 2, 39% coronary disease, 22% TIA or stroke, 18% impaired renal function. At baseline the calcification score was median (IQR) 527 (55–2253) and at 31 months follow-up 1355 (167–4235) which amounts to 44% progression per year. The TEMP-study showed us (RCT etidronate/placebo in PXE patients) that etidronate completely halted the progression of calcification in all the main vascular beds. To be prudent we aim to find a difference between placebo and etidronate group after 1 year of 22%. Alfa 0.05%; Power 80%; Dropout rate 30%. Per arm 40 patients, total 80 will be included, taken into account a drop out of 25%.

Descriptive data will be presented as categorical (n, %) or continuous (mean ± standard deviation or median and interquartile range when appropriate).

Differences in change in CT calcification mass will be analyzed using unpaired Student’s t-tests or Mann-Whitney-U tests when appropriate.

Differences in change in secondary outcomes will be evaluated in similar manners.

## Discussion

Up to now, calcification of vessels in patients with CLTI has been considered the inert endpoint of vascular disease. This belief is based on cross-sectional studies that show extensive calcification in CLTI patients. Yet this type of study cannot differentiate between cause and effect.

So far, research has shown that calcification of the media is an active metabolic process independent of atherosclerosis. This calcification has shown to prevent remodeling and collateral formation in the cerebral circulation (Luijten et al. 2021) Thus, we believe there are reasons to reverse the present paradigm from ‘vascular disease causes calcification’ into ‘calcification cause vascular disease’. This can be proven in a clinical trial if stopping the calcification process improves the clinical outcome.

Etidronate halts the last step of the calcification process. It stops and even reverses the calcification process in nearly all vascular territories (Bartstra et al. 2020) and it decreases aortic stiffness (Kawahara et al. 2013) It also has an excellent risk profile. Yet whether this drug halts calcifications in CLTI is unknown and it is essential to plan a subsequent clinical study to show that etidronate is effective in clinical relevant endpoints.

The current trial focuses on mechanistic, so-called intermediate endpoints, but our main interest is to gain health benefit. Therefore, we already include secondary endpoints such as mortality, cardiovascular intervention of any type, amputation and quality of life. All participants will be followed during and after the trial for the occurrence of these endpoints. We hope to get our ‘mechanistic’ answer quickly and eventually will be able (after longer follow-up without treatment) to evaluate effects on endpoints that are most relevant for patients. This will provide us with the effect size of the treatment with etidronate in the CLTI group. To truly investigate an actual effect on clinical endpoints a phase 3 trial is needed with substantially larger numbers of participants and longer follow-up. The current proof-of-principle trial is the first step to assess efficacy and safety of etidronate in patients with CLTI.

This study may provide evidence that vascular calcification can be stopped with etidronate and may indicate that this could result in improved clinical outcome in CLTI and cardiovascular disease.

## Data Availability

Not applicable.

## Abbreviations

CLTI: Chronic limb threatening ischaemia
PPi: Inorganic pyrophosphate

## References

[CR1] Bartstra JW, De Jong PA, Kranenburg G, Wolterink JM, Isgum I, Wijsman A, Wolf B, Den Harder AM, Mali WPTM, Spiering W (2020). Etidronate halts systemic arterial calcification in pseudoxanthoma elasticum. Atherosclerosis.

[CR2] Bourron O, Phan F, Diallo MH, Hajage D, Aubert CE, Carlier A, Salem JE, Funck-Brentano C, Kemel S, Cluzel P, Redheuil A, Davaine JM, Massy Z, Mentaverri R, Bonnefont-Rousselot D, Gillery P, Jaisson S, Vermeer C, Lacorte JM et al (2020) Circulating Receptor Activator of Nuclear Factor kB Ligand and triglycerides are associated with progression of lower limb arterial calcification in type 2 diabetes: a prospective, observational cohort study. Cardiovasc Diabetol 19(140). 10.1186/s12933-020-01122-4 10.1186/s12933-020-01122-4 PMC7501627 32948184

[CR3] Evrard S, Delanaye P, Kamel S, Cristol JP, Cavalier E, Arnaud J, Zaoui P, Carlier M, Laville M, Fouque D, Cavalier E, Delanaye P, Cristol J, Bargnoux A, Kamel S, Massy Z, Prié D, Urena-Torres P, Souberbielle J et al (2015) Vascular calcification: from pathophysiology to biomarkers. Clin Chim Acta 438:401–414. 10.1016/j.cca.2014.08.034 10.1016/j.cca.2014.08.034 25236333

[CR4] Fleisch H, Schibler D, Maerki J, Frossard (1965). Inhibition of aortic calcification by means of pyrophosphate and polyphosphates. Nature.

[CR5] Kawahara T, Nishikawa M, Kawahara C, Inazu T, Sakai K, Suzuki G (2013). Atorvastatin, etidronate, or both in patients at high risk for atherosclerotic aortic plaques. Circulation.

[CR6] Konijn LCD, Van Overhagen H, Takx RA, De Jong PA, Veger HT, Mali WP (2020). CT calcification patterns of peripheral arteries in patients without known peripheral arterial disease. Eur J Radiol.

[CR7] Konijn LCD, Takx RA, De Jong PA, Spreen MI, Veger HT, Mali WP, Van Overhagen H (2020). Arterial calcification and long-term outcome in chronic limb-threatening ischemia patients. Eur J Radiol.

[CR8] Konijn LCD, Takx RAP, Mali WPTM, Veger HTC, Van Overhagen H (2021) Different lower extremity arterial calcification patterns in patients with chronic limb-threatening ischemia compared with asymptomatic controls. J Personalized Med 11(6). 10.3390/jpm11060493 10.3390/jpm11060493 PMC8226835 34072908

[CR9] Kranenburg G, Bartstra JW, Weijmans M, De Jong PA, Mali WP, Verhaar HJ, Visseren FL, Spiering W (2016). Bisphosphonates for cardiovascular risk reduction: A systematic review and meta-analysis. Atherosclerosis.

[CR10] Kranenburg G, De Jong PA, Bartstra JW, Lagerweij SJ, Lam MG, Ossewaarde-van Norel J, Risseeuw S, Van Leeuwen R, Imhof SM, Verhaar HJ, De Vries JJ, Slart RH, Luurtsema G, Den Harder AM, Visseren FL, Mali WP, Spiering W (2018). Etidronate for prevention of ectopic mineralization in patients with pseudoxanthoma Elasticum. J Am Coll Cardiol.

[CR11] Lanzer P, Boehm M, Sorribas V, Thiriet M, Janzen J, Zeller T, St Hilaire C, Shanahan C (2014). Medial vascular calcification revisited: review and perspectives. Eur Heart J.

[CR12] Luijten SP, Van der Donk SC, Compagne KC, Yo LS, Sprengers ME, Majoie CB, Roos YB, Van Zwam WH, Van Oostenbrugge R, Dippel DW, Van der Lugt A, Roozenbeek B, Bos D (2021). Intracranial carotid artery calcification subtype and collaterals in patients undergoing endovascular thrombectomy. Atherosclerosis.

[CR13] O’Neill WC, Han KH, Schneider TM, Hennigar RA (2015). Prevalence of nonatheromatous lesions in peripheral arterial disease. Arterioscler Thromb Vasc Biol.

[CR14] Oliveira JRM, Oliveira MF (2016) Primary brain calcification in patients undergoing treatment with the biphosphanate alendronate. Sci Rep 6(1). 10.1038/srep22961 10.1038/srep22961 PMC4792151 26976513

[CR15] Rennenberg RJMW, Kessels AGH, Schurgers LJ, Van Engelshoven JMA, De Leeuw PW, Kroon AA (2009). Vascular calcifications as a marker of increased cardiovascular risk: A meta-analysis. Vasc Health Risk Manag.

[CR16] Soor GS, Vukin I, Leong SW, Oreopoulos G, Butany J (2008). Peripheral vascular disease: who gets it and why? A histomorphological analysis of 261 arterial segments from 58 cases. Pathology.

[CR17] Spreen MI, Gremmels H, Teraa M, Sprengers RW, Verhaar MC, Statius Van Eps RG, De Vries JPP, Mali WP, Van Overhagen H (2016). Diabetes is associated with decreased limb survival in patients with critical limb ischemia: pooled data from two randomized controlled trials. Diabetes Care.

[CR18] Teraa M, Conte MS, Moll FL, Verhaar MC (2016) Critical limb ischemia: current trends and future directions. J Am Heart Assoc 5(2). 10.1161/jaha.115.002938 10.1161/JAHA.115.002938 PMC4802465 26908409

[CR19] Van Reijen NS, Hensing T, Santema TKB, Ubbink DT, Koelemay MJ (2021). Outcomes of conservative treatment in patients with chronic limb threatening Ischaemia: A systematic review and Meta-analysis. Eur J Vasc Endovasc Surg.

